# Evaluation of a Pilot Implementation to Integrate Alcohol-Related Care within Primary Care

**DOI:** 10.3390/ijerph14091030

**Published:** 2017-09-08

**Authors:** Jennifer F. Bobb, Amy K. Lee, Gwen T. Lapham, Malia Oliver, Evette Ludman, Carol Achtmeyer, Rebecca Parrish, Ryan M. Caldeiro, Paula Lozano, Julie E. Richards, Katharine A. Bradley

**Affiliations:** 1 Kaiser Permanente Washington Health Research Institute, 1730 Minor Ave, Ste 1600, Seattle, WA 98101, USA; lapham.g@ghc.org (G.T.L.); oliver.m@ghc.org (M.O.); ludman.e@ghc.org (E.L.); achtmeyer.c@ghc.org (C.A.); lozano.p@ghc.org (P.L.); richards.je@ghc.org (J.E.R.); Bradley.k@ghc.org (K.A.B.); 2 Center of Excellence in Substance Abuse Treatment and Education, VA Puget Sound Health Care System 1660 S. Columbian Way, Seattle, WA 98108, USA; 3 Kaiser Permanente Washington, 1200 SW 27th St., Renton, WA 98057, USA; parrish.r@ghc.org (R.P.); caldeiro.r@ghc.org (R.M.C.)

**Keywords:** alcohol drinking, prevention, alcohol use disorders, primary care

## Abstract

Alcohol use is a major cause of disability and death worldwide. To improve prevention and treatment addressing unhealthy alcohol use, experts recommend that alcohol-related care be integrated into primary care (PC). However, few healthcare systems do so. To address this gap, implementation researchers and clinical leaders at Kaiser Permanente Washington partnered to design a high-quality Program of Sustained Patient-centered Alcohol-related Care (SPARC). Here, we describe the SPARC pilot implementation, evaluate its effectiveness within three large pilot sites, and describe the qualitative findings on barriers and facilitators. Across the three sites (*N* = 74,225 PC patients), alcohol screening increased from 8.9% of patients pre-implementation to 62% post-implementation (*p* < 0.0001), with a corresponding increase in assessment for alcohol use disorders (AUD) from 1.2 to 75 patients per 10,000 seen (*p* < 0.0001). Increases were sustained over a year later, with screening at 84.5% and an assessment rate of 81 patients per 10,000 seen across all sites. In addition, there was a 50% increase in the number of new AUD diagnoses (*p* = 0.0002), and a non-statistically significant 54% increase in treatment within 14 days of new diagnoses (*p* = 0.083). The pilot informed an ongoing stepped-wedge trial in the remaining 22 PC sites.

## 1. Introduction

Alcohol use accounts for 3.6% of mortality and 4.6% of disability worldwide [1]. Unhealthy alcohol use includes a spectrum from drinking above recommended limits to alcohol use disorders (AUD) [2], and can result in multiple severe health problems, including trauma, cirrhosis, cancer, and poor management of other chronic diseases [1,3,4].

Several options for evidence-based prevention and treatment are available to address unhealthy alcohol use. Routine alcohol screening and brief alcohol counseling (“brief intervention”) for patients who screen positive for unhealthy alcohol use decrease drinking and have been found to be cost effective [5,6,7,8,9,10,11,12]. For AUD treatment, evidence and guidelines support medications and counseling (e.g., motivational enhancement therapy, cognitive behavioral therapy), and specialty alcohol treatment [13,14,15,16,17,18,19,20,21,22,23]. However, most patients do not receive alcohol screening and brief intervention [24] because sustained implementation has been challenging. Further, most patients with AUD never receive treatment either because AUDs remain unrecognized or because they do not accept referral [25], and they are less likely to receive appropriate treatment than patients with any other common chronic disease [26].

To address this gap, experts recommend that alcohol-related care be integrated into primary care (PC) similar to care for other common chronic conditions, such as diabetes and depression [27,28,29,30]. Yet, despite several exceptions [31,32,33,34], few healthcare systems have successfully implemented alcohol screening: screening rates are low (2–26%) and poorly maintained over time [35]. Providing high-quality, population-based alcohol-related medical care is challenging due to several barriers, including stigma, a lack of training, not having alcohol use on the agenda of prevention topics in PC, not seeing AUDs as within the scope of conditions treatable within PC, and the perception of a single treatment option for AUD in the U.S.; namely, abstinence-oriented, group-based rehabilitation based on the 12 steps of Alcoholics Anonymous (AA) [36,37,38,39]. The Program of Sustained Patient-centered Alcohol-related Care (SPARC) was designed to address these barriers.

Implementation researchers and clinical leaders at Kaiser Permanente (KP) Washington partnered to design the SPARC program in 2013 in response to a call for research proposals focused on the sustainable implementation of evidence-based practices from the U.S. Agency for Healthcare Quality and Research (AHRQ). The SPARC implementation strategy was built on the successes and limitations learned from implementing alcohol screening and brief intervention across 21 Veterans Affairs networks in over 900 clinical sites nationwide [31,40]. Despite widespread implementation, there were marked gaps in the quality of alcohol screening [41], and documented brief interventions increased more than patient-reported alcohol-related counseling [42,43]. SPARC applied state-of-the-art quality improvement approaches with a participatory design to increase provider and staff engagement, used patient self-report on paper screens to increase the quality of screening, and focused on increasing provider comfort in providing alcohol-related care by explicitly addressing stigma, a critical barrier to evidence-based alcohol-related care. The partnership between researchers and clinical leaders began in 2012, when health system leaders invited researchers to conduct an evaluation of the current state of addictions care. The evaluation identified important gaps in the access to and coordination of care for alcohol and other substance use disorders, and increased the motivation of health system leaders to address the gaps. The resulting SPARC program aimed to implement sustained, high-quality alcohol-related care in all 25 PC sites in KP Washington. After the proposal was funded by AHRQ, KP Washington leaders requested that the grant be used to support the integration of behavioral health care more broadly into PC. Behavioral Health Integration (BHI) included the screening, assessment, diagnosis, treatment, and monitoring of depression symptoms [44,45,46], unhealthy alcohol use [47], cannabis use, [48], illicit drug use, and/or non-medical use of prescription medication [49]. A pilot in three large PC sites (SPARC pilot) was conducted before proceeding to conduct a full stepped-wedge pragmatic trial in the remaining 22 sites (SPARC trial).

In this paper, we provide an overview of the SPARC pilot and the implementation strategies used to integrate alcohol-related care within PC. We quantitatively evaluate the effectiveness of the SPARC implementation by comparing the outcomes before versus after implementation within the three pilot sites. We hypothesized that the introduction of SPARC would be associated with increased rates of screening for unhealthy alcohol use, the assessment for AUD, and the new diagnosis and treatment of AUD. Additionally, we qualitatively describe barriers and facilitators to providing alcohol-related care that were encountered during the SPARC pilot, which led to changes in SPARC implementation strategies for the subsequent full pragmatic trial.

## 2. Materials and Methods

### 2.1. Setting

**Figure 1:**
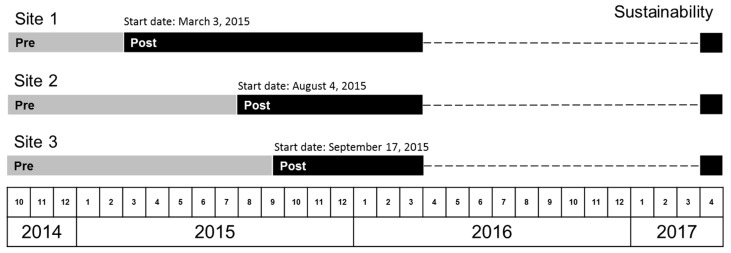
Pre- and post-implementation periods for Sites 1–3.

The SPARC pilot took place at three PC sites in KP Washington, an integrated health care delivery system and insurance plan in Washington State (formerly Group Health Cooperative in Washington State, USA). These three sites were selected by health system leaders due to site leaders’ receptivity to behavioral health integration and geographic representation in western Washington. They were large outpatient medical centers, with two to four separate PC clinics at each site, located in different wings or floors of the building. Site 1 had an urgent care clinic on site. Sites 1 and 2 had specialty mental health clinics on site. Our study population consisted of all patients (ages 18 or older) who had an in-person visit to a PC provider in one of the three pilot sites during the pilot study period. The pilot study period (see timeline in Figure 1) was from 3 October 2014, 5 months prior to the official launch of SPARC at the first site, until completion of pilot activities on 1 April 2016, when the roll-out of the SPARC program began in a pragmatic stepped-wedge trial in the other 22 KP Washington PC sites.

### 2.2. The SPARC Program

SPARC included evidence-based alcohol-related prevention [5,6] and treatment [13,14,15,16,17,18,19,20,21,22,23]. The target was to screen 80% of PC patients annually for unhealthy alcohol use with the validated Alcohol Use Disorders Identification Test–Consumption questionnaire (AUDIT-C), offer brief intervention to 80% of patients who screened positive, including recommendations that patients drink below the recommended U.S. limits [50], and provide feedback linking unhealthy alcohol use to specific health conditions of interest to the patient (e.g., hypertension, insomnia, and breast cancer prevention) [51]. In addition, components of the SPARC program aimed at improving the quality of care for AUD, which included assessing 80% of patients with high-risk unhealthy alcohol use for Diagnostic and Statistical Manual of Mental Disorders (DSM-5) AUD symptoms, and the diagnosis and treatment of “new” AUD (not documented in the prior year) [52]. Targets were set at 80% to allow clinicians the autonomy to prioritize clinical activities in a particular visit and encourage appropriate care when other issues were more important or screening was not appropriate (e.g., cognitively impaired patients).

### 2.3. SPARC Implementation Strategies

The approach used to implement SPARC addressed several of the barriers to high-quality alcohol-related care mentioned above by using three main strategies (Table 1): (1) enabling PC teams to offer high-quality alcohol-related care by addressing stigma, providing training, designing and implementing workflows to deliver the care, promoting buy-in, and addressing other barriers; (2) supporting PC teams through electronic health record (EHR) decision support, including prompts and other EHR tools to cue the delivery of brief intervention and assessment of AUD symptoms; and (3) systematic performance monitoring of screening and assessment rates with active feedback to clinic leaders and teams. SPARC was implemented with BHI more broadly; the study supported the development of EHR tools and performance measures for all elements of BHI. Details are shown in Table 1.

### 2.4. Planned Timeline of Implementation

Although the three pilot sites originally were expected to implement SPARC at the same time, the timeline was renegotiated due to health system reorganization and leadership transitions, resulting in three separate start dates (Site 1: 3 March 2015; Site 2: 4 August 2015; and Site 3: 17 September 2015). These negotiated start dates were used in the quantitative analyses (described below) as the “official” start dates of the SPARC implementation at each site (see Figure 1). Prior to the implementation start date, each pilot site participated in a 3-day design event to develop, pilot, and refine the workflow for the SPARC trial. The design events occurred within a week before the official start dates at all three pilot sites.

### 2.5. Quantitative Metrics

We evaluated whether the implementation of SPARC led to changes in the rates of: screening for unhealthy alcohol use; assessment with the use of an AUD Symptom Checklist created for this project by clinical leaders; and documentation of new diagnoses and treatment of AUD. All metrics were defined using routinely collected EHR data. Although brief intervention for unhealthy alcohol use was an important aspect of the SPARC pilot, we did not evaluate whether the implementation successfully led to changes in documented brief intervention in the pilot. Clinical leaders decided to encourage brief interventions without specific requirements or monitoring of documentation to focus clinicians on getting comfortable with brief counseling due to concerns about possible “over-documentation” in previous implementation efforts [42]. For each PC site, we identified whether patients were screened, assessed, newly diagnosed with AUD, and treated for new AUD during the pre- and post-implementation pilot periods, using each sites’ negotiated implementation start date. Measures were defined as follows.

#### 2.5.1. Alcohol Screening

Prior to the implementation of SPARC, there were several different mechanisms by which a PC patient might have been screened in PC (e.g., through a well-visit questionnaire with the 3-item AUDIT-C or via ad hoc screening when a PC provider was concerned). In addition, the AUDIT-C was a part of a routine questionnaire in behavioral health clinics as well as in urgent care for patients presenting with behavioral health concerns.

After implementation of SPARC in the context of BHI, the AUDIT-C could additionally be completed as part of a 7-item behavioral health paper questionnaire filled out by the patient and entered into the EHR by the medical assistant (including also a 2-item depression screen [55] and two items for screening for cannabis and drug use [49]). A screen was positive for unhealthy alcohol use if the AUDIT-C was ≥ 3 for women and ≥ 4 for men, and AUDIT-C scores ≥ 7 indicated high-risk unhealthy alcohol use [56,57,58] that required assessment with the AUD Symptom Checklist.

For each PC visit, we identified whether the patient was screened for unhealthy alcohol use on the day of the visit or within the prior year to reflect the goal that patients would be screened annually. We note that under this metric, if a patient was screened in the pre-implementation period, then that patient is considered as having been compliant for annual screening at any subsequent visit up to one year following the screening event (including visits in the post-implementation period). Because the same screen was used in urgent care and the behavioral health clinic, PC patients were not re-screened if they had already been screened elsewhere.

#### 2.5.2. Assessment for DSM-5 AUD Symptoms

As part of SPARC, an 11-item AUD Symptom Checklist, based on DSM-5 AUD [59], was developed for patients to fill out and medical assistants to enter into the EHR. At each PC visit, we first identified the patient’s most recent alcohol screen, either from the day of the visit or from the prior year (if done). An assessment was considered needed if the screening score indicated high-risk unhealthy alcohol use indicating increased risk for AUD (AUDIT-C score ≥ 7 points) [56,57,58]. Among patients needing an assessment, we determined whether the patient was assessed for AUD, indicated by EHR documentation of the 11-item Symptom Checklist, within a year prior to the high-risk AUDIT-C or within the time from the high-risk screen up to and including the visit date. As with the screening metric, the assessment could occur anywhere within the health system (not solely within PC).

#### 2.5.3. New AUD Diagnosis and Treatment

At each PC visit, we identified whether the patient had a new AUD diagnosis on the date of the visit. A new AUD diagnosis was defined in this study as an AUD diagnosis without any prior AUD diagnosis within the past year, using International Classification of Disease (ICD) codes from the U.S. National Committee for Quality Assurance’s (NCQA’s) Healthcare Effectiveness Data and Information Set (HEDIS) measure for the Initiation and Engagement of Alcohol and Other Drug Dependence Treatment [52]. If the patient had a new AUD diagnosis on that visit, we identified whether the patient was treated for AUD or other substance use disorders within the following 14 days based on data from the EHR or claims for outside care (but not including AA). Treatment was defined as a follow up visit for AUD based on International Classification of Diseases (ICD) codes, again using the HEDIS definition of initiation of AUD treatment [52]. Of note, this pilot study did not obtain data on the exact care provided, which could have ranged from PC counseling or medications for AUDs from PC or mental health clinics, to specialty addictions treatment outside the health system (provided by contracted providers in the community). Our primary time window of interest was 14 days, consistent with NCQA’s HEDIS measure for treatment initiation [52], but we also considered 30-day and 90-day windows to assess whether SPARC implementation increased the treatment of new AUDs over a longer timeframe.

### 2.6. Statistical Analysis

#### 2.6.1. Descriptive Analyses

Only some of the PC clinics within each site implemented Behavioral Health Integration (see Section 3.1 below). Unless otherwise specified, all analyses were restricted to patients who had made visits to PC providers in a PC clinic that implemented BHI. We will refer to these PC providers as BHI providers. We described the demographic characteristics of patients with at least one PC visit to a BHI provider separately within the pre- and post-implementation periods.

#### 2.6.2. Time Series Analyses

We conducted time series analyses using week intervals for screening and month intervals for assessment, diagnosis, and treatment. Specifically, we constructed visit-based binary indicators of each outcome and calculated the proportion of all PC visits to BHI providers during an interval (week or month), in which the patient (1) was screened for unhealthy alcohol use, (2) needed an AUD assessment and was assessed, (3) had a new AUD diagnosis, or (4) had a new AUD diagnosis and was treated. These proportions were plotted over the study period, separately for each clinic.

Note that all of these main study outcomes have as their denominator the number of PC visits, as opposed to a denominator that could be affected by the implementation, such as patients with positive AUDIT-C screens [60]. This approach was used because the alternative would make the outcome challenging to interpret (since changes to the outcome could be due to changes to the denominator, numerator, or both). For example, integrating routine alcohol-related care likely changes the population of patients who are screened from a selected, high-risk subpopulation to a PC population. Therefore, individuals being screened, assessed, or diagnosed post-implementation would not be comparable to those being screened, assessed, or diagnosed in the pre-implementation period unless all PC patients seen during the time interval are used as the denominator.

#### 2.6.3. Pre-Versus Post-Implementation Analyses

We defined the pre-implementation period as the period prior to the official launch date of SPARC care (as part of BHI) within the site, defined above, and the post-implementation period as the period following the official launch date (see Figure 1). Note that these pre- and post-implementation periods were defined a priori and may not correspond to the actual date when providers began implementing BHI.

Using the first visit of a patient to a BHI provider within the pre- and post-implementation periods, respectively, as above, we calculated the proportion of patients who (1) were screened for unhealthy alcohol use, (2) needed an AUD assessment and were assessed, (3) had a new AUD diagnosis, and (4) had a new AUD diagnosis and were treated. Below, we report proportions either as percentages (e.g., number of patients screened per 100 seen) or as the number per 10,000 patients seen, as appropriate. We tested whether there was a significant difference in these proportions in the pre- versus post- period while accounting for correlation of repeated visits (if the same patient had a visit in both periods) by using generalized estimating equations (GEE) [61]. Specifically, we fit a separate logistic GEE model for each of the visit-specific binary outcomes (e.g., indicator for whether the patient was screened at that visit) regressed on an indicator for whether the visit occurred in the pre- vs. post-implementation period. We used an independent working covariance structure and the robust sandwich variance estimator; P values were calculated using the Wald test. We interpret P values less than 0.05 as statistically significant.

#### 2.6.4. Sustainability

As a measure of sustainability, we also calculated rates of alcohol screening and assessment in April 2017, which was 18, 14, and 13 months after the end of the active pilot implementation at Site 1, Site 2, and Site 3, respectively (see Figure 1). These analyses used the first visit of PC patients to a BHI provider during April 2017 as the denominator.

### 2.7. Implementation-Focused Formative Evaluation

This pilot study did not include formal analyses of qualitative data, but instead we report on the results of the formative evaluation (FE). The two practice coaches interacted with staff in the three pilot PC sites regularly regarding elements of planned implementation that were challenging, and brought information on barriers and facilitators to a weekly implementation-focused FE meeting that included the principal investigator, a co-investigator, and a project manager [62]. This FE meeting, between the coaches and other investigators, was used to identify barriers and facilitators to using the planned implementation strategies and brainstorm approaches to maximize facilitators and overcome barriers. Proposed adaptations were then presented and discussed at the weekly meeting with the entire leadership team: clinical leaders leading BHI and implementation researchers. Detailed minutes of both FE and weekly meetings were taken by a research team member in real-time, for later template coding [63] based on the Greenhalgh model [64]. However, in this report, we summarize those barriers and facilitators that led to changes to the implementation strategies in the subsequent stepped-wedge trial (across the other 22 PC clinics), as well as the resulting changes to implementation strategies (without formal coding of qualitative data).

## 3. Results

### 3.1. Pilot Sites Selected by Health System Leaders

Because support from integrated behavioral health clinicians was limited and felt to be a key ingredient for success for BHI at all design events (Table 1), local PC leaders at all three pilot sites decided to implement BHI only in some of their PC clinics. This was not originally planned. Site 1 implemented in two of three PC clinics, Site 2 implemented in one of two PC clinics, and Site 3 implemented in two of four PC clinics. The PC providers in the clinics implementing BHI are referred to as BHI providers below. At each site, about half of the PC providers implemented BHI. Additionally, while most BHI providers at Sites 1 and 3 began implementing BHI within a month of the original start date, BHI providers at Site 2 did not begin implementing BHI until 3 months after the original start date.

### 3.2. Study Sample

There were 53,133 patients with a visit to a BHI provider during the study period, of whom 32,295 had a visit in the pre-implementation period and 39,599 had a visit in the post-implementation period (18,761 had a visit in both periods). The number of visits per patient across the study period ranged from 1 to 69, with a mean (interquartile range (IQR)) of 2.57 (1, 3) visits. Patients with a visit to a BHI provider in the pre-implementation period were predominantly female (62.0%), non-Hispanic (92.2%), white (82.3%), and had a mean (IQR) age of 54.5 (40, 68) years. These demographic characteristics were similar among patients with a visit in the post-implementation period, though statistically significantly different due to large sample sizes (Table 2).

### 3.3. Quantitative Comparison of Care before versus after Implementation

**Figure 2:**
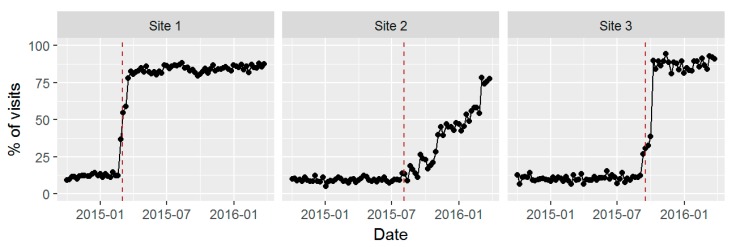
Screening for unhealthy alcohol use: percentage (%) of visits with screening by week, among providers implementing Behavioral Health Integration including the Sustained Patient-centered Alcohol-related Care (SPARC) program within three PC sites.

**Figure 3:**
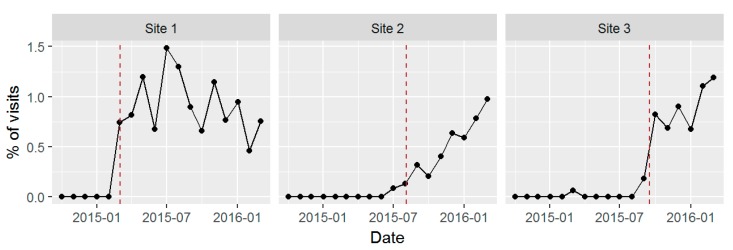
Assessment for alcohol use disorders (AUD): percentage (%) of visits with completed AUD Symptom Checklist by month, among providers implementing Behavioral Health Integration including the Sustained Patient-centered Alcohol-related Care (SPARC) program within three PC sites.

**Figure 4:**
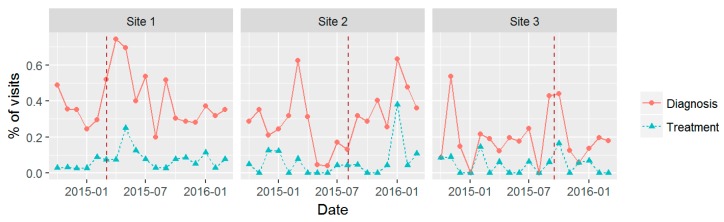
New diagnosis and treatment for alcohol use disorders (AUD): percentage (%) of visits by month, among providers implementing Behavioral Health Integration including the Sustained Patient-centered Alcohol-related Care (SPARC) program within three PC sites.

Figure 2, Figure 3 and Figure 4 show time series plots of the rates of the primary study outcomes over the course of the study period separately in each of the three pilot sites. Table 3 shows the overall rates of these outcomes in the post- versus pre-implementation periods, pooled across the three sites.

#### 3.3.1. Alcohol Screening

Although many approaches to screening were used before BHI, only 8.9% of PC patients seen before BHI had been screened. The proportion of weekly visits in which patients were screened for unhealthy alcohol use increased dramatically in the few weeks following the official launch date in Sites 1 and 3, while there was a slower rise in the proportion of visits in which patients were screened at Site 2 (Figure 2). From the week before launch to week 4 after the official launch, the percentage of visits in which the patients were screened increased from 12.4% to 82.4% in Site 1, from 9.1% to 16.6% in Site 2, and from 12.1% to 89.9% in Site 3. At Site 2, local leadership decided to identify additional provider champions to continue piloting the work before rolling out to the rest of the BHI providers due to concerns about increased workload negatively impacting provider morale.

Across sites, the overall percentage of patients who were screened at their first visit increased from 8.9% in the pre- period to 62% post-implementation (*p* < 0.0001; Table 3). Comparing pre- and post-implementation, SPARC also increased the prevalence of documented unhealthy alcohol use (AUDIT-C scores ≥3 for women and ≥4 for men) from 2.2% to 17% (*p* < 0.0001), and the prevalence of documented high-risk unhealthy alcohol use (AUDIT-C scores 7–12) from 0.31% to 1.4% (*p* < 0.0001).

#### 3.3.2. Assessment for DSM-5 AUD Symptoms

The percentage of visits to BHI providers in which patients were assessed for AUD increased steeply around the time of the official launch date and then leveled off or slightly declined at Site 1, increased gradually at Site 2 following the launch date, and increased sharply following launch at Site 3 (Figure 3). Across all sites, the proportion of PC patients who both needed an AUD assessment and were assessed increased from 1.2 patients per 10,000 seen in the sites before BHI to 75 per 10,000 (*p* < 0.0001; Table 3). The number of patients with a positive assessment (two or more symptoms of AUD on the Symptom Checklist) increased from 0.62 to 40 patients per 10,000 PC patients seen (*p* < 0.0001); Table 3 shows the same results as percentages.

#### 3.3.3. New AUD Diagnosis and Treatment

There was not a clear pattern in the time series of the number of patients with visits to BHI providers with a new AUD diagnosis or who were treated for AUD within the following 14 days (Figure 4). Across all sites, the number of patients with a new AUD diagnosis at their first visit during the period increased from 39 per 10,000 patients in the pre-implementation period to 58 per 10,000 in the post-implementation period (*p* = 0.0002; Table 3). This increase was driven by Site 1, where the number of patients newly diagnosed increased from 42 to 75 per 10,000 (*p* = 0.0003); in the other two sites, there was no difference in the number of patients per 10,000 with a new diagnosis (from 44 to 42 in Site 2 (*p* = 0.79) and from 28 to 30 in Site 3 (*p* = 0.85)).

Using our primary treatment measure, there was not a statistically significant difference between pre- and post-implementation in the number of patients with a new AUD diagnosis who were treated within the following 14 days (*p* = 0.083; Table 3), the timeframe of the HEDIS treatment initiation measure. However, broadening the definition to allow for treatment initiation over a longer timeframe did reveal statistically significant increases associated with BHI. In particular, the number of patients with a new AUD diagnosis and treatment increased from 8.7 to 14 per 10,000 under the 30-day metric (*p* = 0.034) and from 11 to 18 per 10,000 under the 90-day metric (*p* = 0.024). As with the new diagnosis rates, this increase in treatment for AUDs was driven by Site 1 (results not shown).

#### 3.3.4. Sensitivity Analyses

Our above analyses were restricted to BHI providers, as only these providers were selected by the site leaders to implement BHI. As a sensitivity analysis, we included patients of all providers (including those who did not implement BHI). This attenuated our estimates of the change in screening, assessment, new diagnosis, and treatment rates as expected, though estimates remained statistically significant (except for the 30-day treatment outcome). As a second sensitivity analysis, instead of using the first visit of the patient during both the pre- and post- periods, we used the last visit. This led to larger estimates of the change in screening and assessment rates (since using the last visit allows for more time for the intervention to go into effect), but slightly smaller estimates of the change in new diagnosis rates (since by the last visit, more people had already had a new diagnosis earlier in the period, and so could no longer be newly diagnosed).

### 3.4. Sustained Screening after Active Support for Implementation Ended

In April 2017, among those PC patients with a visit to a BHI provider, 84.5% completed alcohol screening across the three sites, with site-specific rates of screening of 81.8%, 84.8%, and 89.6% for Site 1, Site 2, and Site 3, respectively; in addition, 0.81% (81 per 10,000) completed a Symptom Checklist for AUD overall, with site-specific rates of 0.54%, 1.31%, and 0.71%, respectively.

### 3.5. Results of Formative Evaluation

In this section we describe qualitative findings from the implementation-focused formative evaluation that led to a change in the implementation strategies in the full stepped-wedge pragmatic trial in the remainder of the 22 PC clinics. Implementation in the pilot sites was carried out consistently with planned approaches with the exception of the elements described below, which were modified or enhanced during the pilot.

#### 3.5.1. Implementing BHI Did Not Lengthen Patient Visit Time

Although initially complex for medical assistants to learn, during the piloting stages of the design events (days 2 and 3), medical assistants quickly developed approaches to efficiently ask patients to complete the 7-item screen and the necessary assessments depending on screening results and EHR prompts. PC clinicians found that having the results of screening and assessment before they entered the room, as well as the availability of integrated behavioral health clinicians to aid in brief alcohol counseling for patients with AUDIT-Cs over 6 or AUDs, increased efficiency. As a result, implementation did not lengthen the total patient visit time (which included both time with the medical assistants and with providers) during the design event in Site 3, and during the design events in Sites 1 and 2, the total patient visit time actually decreased by 4 to 7 min. This helped increase receptivity to annual screening and BHI.

#### 3.5.2. Need for Active Practice Facilitation

Initially at Sites 1 and 2, practice facilitation was relatively passive with the sites choosing how often to meet to address gaps in achieving BHI targets. After 6 months of implementation at Site 1, and a slow launch at Site 2, a decision was made to more actively facilitate implementation and the practice coaches were trained through the Dartmouth Institute Microsystem Academy’s approach to clinical microsystem improvement [65]. After this change, weekly meetings were scheduled with an interdisciplinary local implementation team at Site 3, and eventually every other week at Site 2, to facilitate rapid cycle improvements. The PC site managers, PC provider champions, medical assistant champion(s), and PC social worker(s) involved in the design events became part of each site’s local implementation team, which met weekly to monthly, depending on the site, and reviewed data to identify and problem-solve challenges. Local implementation team members became experts within their sites in BHI and helped support peer-to-peer learning.

#### 3.5.3. Immense Value of Using Stories to Increase Engagement

Local stories, such as one at Site 1, had a profound effect on PC teams implementing BHI. A Site 1 physician leader, who was skeptical about the benefits of BHI, had been caring for a patient with diabetes and severe vascular disease who had a lower extremity amputation, and was being seen weekly for lower extremity ulcers. At the start of BHI implementation, the EHR prompted the medical assistant to ask the patient to complete the BHI screen with the AUDIT-C, and then (because he had a high-risk AUDIT-C) the 11-item AUD Symptom Checklist. The medical assistant gave the information to the physician who then met with the patient and discovered he was drinking a fifth of vodka a day. As a result of the discussion with the physician, and then a more in-depth assessment with a social worker, the patient initiated care for his alcohol use disorder that day and left with a care plan. Stories like these that highlighted the patient benefit and value in alcohol-related care in PC, helped increase the engagement in and adoption of BHI (and consequently SPARC) in the PC teams. For the full trial, we explicitly use stories to foster optimism, self-efficacy, and ownership of alcohol-related care.

#### 3.5.4. Implementing SPARC Clinical Care in the Context of BHI May Have Facilitated Adoption

Although the SPARC clinical care was not originally designed as part of a larger BHI initiative, the framing of BHI as “whole person care” to ensure that there is “no wrong door” for people to start getting help for their behavioral health conditions may have facilitated the adoption of alcohol-related care in PC. For instance, the inclusion of depression care and a standardized assessment of patients with suicidal ideation led to many positive stories about improved patient care due to BHI, which may have increased receptivity and influenced attitudes in adopting SPARC.

#### 3.5.5. Training Social Workers to Manage Addictions in PC before BHI Implementation

By chance, leaders had also assigned Site 1 to participate in another quality improvement project testing the feasibility of the population-based management of patients with AUD or other substance use disorders by PC social workers. This other Partnership in Innovation project funded by Group Health Foundation began in December 2014 [54]. The project—called the 3:30 Project because it was designed to bring patients with new alcohol or substance use disorders diagnoses for three visits addressing alcohol and substance use disorders within 30 days—engaged patients in addictions care management by social workers. Social workers used an EHR registry, pro-active outreach, assessment, motivational interviewing (MI) skills, shared decision-making about evidence-based treatment options, and referral to treatment as appropriate. Social workers were trained for 4 h regarding evidence-based treatment options for addictions and using MI skills and shared decision-making to engage and motivate patients [29,66]. Social workers were supervised weekly for an hour by a four-person interdisciplinary team (addictions psychiatrist, psychologist with MI expertise, PC physician, and social work manager). One of two social workers at Site 1, who had little previous addictions experience, reported markedly increased self-efficacy for engaging patients with addictions in PC. The other social worker had prior addictions treatment experience and so was already comfortable. When launching BHI 3 months later, the social workers expressed comfort working with patients with alcohol or high-risk use substance use, and welcomed “warm hand-offs” from PC providers. PC providers in Site 1 reported that the expertise of the social workers increased their comfort with BHI as well as working with the social workers to partner in caring for patients.

As a result, the 3:30 training and supervision were “rolled out” to all PC social workers in preparation for BHI. In addition, the social workers’ roles as integrated behavioral health clinicians, including providing patients on-site, short-term behavioral health care in PC, has been so central to the perceived success of BHI by clinicians and leaders that social work staffing is being increased across the system to meet BHI needs, and all social workers are using the 3:30 EHR registry and attending weekly addictions supervision meetings.

#### 3.5.6. Development of an Alcohol Video to Address Stigma

The original proposal for SPARC included an alcohol handout that was designed to address stigma (available as Supplemental Material). We were then able to obtain funding to make a public health video with Dr. Mike Evans on alcohol and health (https://youtu.be/tbKbq2IytC4) [53]. Both were designed to overcome patient and provider stigma regarding unhealthy alcohol use. PC provider champions noted that the video was very helpful in understanding the focus of patient-centered care for unhealthy alcohol use, and they mimicked the video in developing scripts for patients. As a result the video has been used, along with the handout, in training all PC staff for BHI.

#### 3.5.7. Anecdotal Increases in Staff Satisfaction

Staff satisfaction was one benefit of the BHI program overall that facilitated the implementation of SPARC. One provider from Site 2 expressed appreciation for the ability to systematically address common behavioral health conditions in PC, especially with added support from social workers, and that BHI has helped changed the culture in PC. In Site 3, a medical assistant, who rooms patients and administers and enters all screening and assessments, talked about how he felt empowered as he learned new skills in engaging with patients around behavioral health conditions. He reported that the medical assistants saw first-hand how BHI opened the door for patients to talk about their overall health in a non-stigmatizing environment.

## 4. Discussion

Among the PC clinics selected by site leaders of KP Washington to implement SPARC, we found a large increase in the rates of screening for unhealthy alcohol use in the post-implementation period compared to the pre-implementation period. The increase in screening rates was accompanied by corresponding increases in the rates of assessment for AUD across the three sites. There was an overall 50% increase in the number of new diagnoses for AUD (from 0.39% to 0.58% of PC patients) across the three sites. The rates of treatment within 14 days of a new AUD diagnosis increased by 54% (from 0.065% to 0.10% of PC patients); although this change was not statistically significant, there was a significant increase in treatment rates within 30 and 90 days of a new diagnosis. Additionally, we found that the increases in screening and assessment were sustained after active implementation ended. Moreover, anecdotally, both staff and patients liked the program; leaders of KP Washington decided to implement the program across the other 22 clinics as a result.

Although evidence-based care for unhealthy alcohol use includes preventive screening, brief intervention, diagnosis, and treatment of AUDs [5,6,7,8,9,10,11,12,13,14,15,16,17,18,19,20,21,22,23], relatively little research has focused on whether population-based screening can increase the diagnosis and treatment of AUDs. In the 1970s and 1980s, before seminal studies of brief intervention in PC [67], alcohol screening was focused on improving the low rates of identification of AUD in PC [68,69], and PC experts recommended that the patient then be told the diagnosis and advised to abstain and seek treatment [70]. Although one study found that informing providers of their patients’ symptoms of DSM AUD increased counseling [71], we know of no prior study to evaluate whether population-based screening was associated with increased diagnosis and/or treatment of AUDs, which will be evaluated in the full stepped-wedge trial.

This pre-post study used a standardized AUD Symptom Checklist to assess patients with high-risk AUDIT-C scores. The findings suggest that population-based screening, with the further assessment of symptoms, may increase both diagnosis and treatment. However, although we found an overall increase in the rates of new diagnosis and in the 30-day and 90-day treatment metrics, this change was driven by just one of the sites (Site 1). The findings might have been driven by a Site 1 social worker with prior addictions experience or potentially driven by Site 1’s involvement in the 3:30 training and supervision program to engage patients in addictions care management by PC social workers. Site 1 was also a very large clinic and was followed for much longer after implementation. In addition, Site 2 had a very slow increase in DSM-5 assessments for AUDs, likely as a result of their gradual implementation of screening. While Site 3 had rates of assessment comparable to Site 1, Site 3 was followed for a shorter period after implementation. It is also important to note that these three sites were selected by leaders for this pilot in part based on their anticipated receptivity to BHI.

Despite a significant increase in AUD diagnosis and treatment, only 0.4% of patients had two or more DSM-5 AUD symptoms (consistent with mild AUD), and only 0.6% of patients had a new AUD diagnosis documented. Given that the prevalence of an AUD diagnosis in the U.S. is estimated to be 13.9% [72], it will be important for the full trial to evaluate the overall proportion of PC patients with AUDs (both previously and newly recognized) to estimate the magnitude of continued unrecognized AUDs.

A pragmatic stepped-wedge trial of SPARC is currently underway to systematically evaluate the effectiveness of the SPARC implementation in the remaining PC sites (ClinicalTrials.gov Identifier: NCT02675777) and will address many of the limitations of the current analysis. The results of this ongoing stepped-wedge trial in the 22 other PC sites in this system will determine whether the program of population-based screening, with the use of a Symptom Checklist to assess high-risk patients, as well as the full range of SPARC implementation strategies, which are being iteratively improved upon, along with weekly social work supervision on the use of a registry and management of addictions care in PC, increases diagnosis and treatment.

Within each pilot site, the implementation was not adopted by all providers by the end of the pilot period; rather, local site leaders selected a subset of clinics to implement BHI. However, within 11–17 months of the official launch dates, each site rolled BHI out to all of its clinics. The qualitative findings from the formative evaluation suggest that several factors contributed to the success of implementing SPARC within the larger BHI initiative and in the increasing adoption of evidence-based care for unhealthy alcohol use and AUDs. Local leadership at the three pilot sites decided that the BHI model, which included the SPARC alcohol-related care, was beneficial for their patients and needed to be expanded to provide all PC patients high-quality behavioral health care throughout their site. The larger health system helped support expanding BHI to the remaining clinics by adding social work support. Overall, the early inclusion of frontline staff in the designing of workflow processes, the use of practice coaches to work with site teams to do rapid cycle improvements, the use of patient stories to increase engagement, and the inclusion of SPARC clinical care in the broader context of BHI with social worker support were all facilitators to the implementation of SPARC.

Our analysis of the changes in alcohol measures pre- versus post-implementation was limited to the three pilot sites, which were selected by health system leaders and thus may represent sites more open to implementing evidence-based alcohol-related care. Thus, our estimates of the changes in outcomes in these particular sites may not be reflective of what might be expected to occur at the remaining sites that were not selected as pilot sites. Additionally, we used a pre–post analysis. Although our estimates of the increase in screening and assessment rates were quite large and unlikely to be due to other factors that changed over the same time period, the changes in the rarer outcomes of new diagnosis and treatment of AUDs may reflect concurrent time trends. It is not clear that the implementation of SPARC alone would have been successful if it were not integrated with the implementation of care for other common behavioral health conditions. Finally, brief intervention was not measured in this pilot because clinical leaders did not require PC providers to document brief intervention as part of their progress notes. For the main pragmatic trial, codes for screening and brief intervention were incorporated into the PC provider training materials. We will also use natural language processing to extract standardized text about brief alcohol-related counseling from progress notes.

## 5. Conclusions

The approach implemented by KP Washington to integrate alcohol-related care as part of behavioral health integration increased alcohol screening and assessment rates for AUD, and appeared to increase rates of new AUD diagnosis and treatment. The full pragmatic trial will evaluate this approach across sites that were not selected as “early adopters” by clinical leaders. Furthermore, randomization of implementation start dates will provide stronger evidence as to a causal link between the SPARC implementation and changes in alcohol-related care.

## Figures and Tables

**Table 1:** The Program of Sustained Patient-centered Alcohol-related Care (SPARC): Components of the Implementation Strategy.

**Strategy #1: Enabling Primary Care (PC) Teams to Offer High-Quality Alcohol-Related Care**
Components:
Training of PC teams. Recruited PC provider champions from each site, and trained them with social workers (8 h). Front line design. A 3-day design event led by a KP Washington Lean process improvement consultant involved front line staff—an interdisciplinary local implementation team of PC provider, medical assistant, nurse, and social worker champions—in designing implementation and workflows, with iterative improvement of workflows in days 2 and 3 while piloting in real time with patients. At the end of the design events, local implementation teams, local site leaders, health system leadership, and researchers discussed plans for the next steps of implementation, including needed training and launch dates. Support from practice coaches. An external practice coach worked with PC teams to support implementation and quality improvement. Learning sessions. Every other week, PC provider champions participated in learning sessions: teleconferences with the external practice coach and health system and research experts on screening and brief intervention, alcohol use disorders (AUD) treatment, shared decision-making, and motivational interviewing (MI). Learning sessions encouraged champions to share stories and problem-solve challenges, and supported provider-to-provider exchanges. For patients with AUDIT-C scores of 3–6 (women) or 4–6 (men), brief interventions were framed as brief preventive advice with the use of an alcohol handout (see Supplemental Materials), “for all patients who drink regularly”, where PC providers would review recommended drinking limits, link alcohol to health concerns, and elicit patient response. For patients with AUDIT-C scores of 7–12, PC providers were asked to introduce the patient to an integrated behavioral health clinician (described below) for brief alcohol counseling guided by principles of shared decision-making and results from the AUD Symptom Checklist (described in Section 2.5.2). Addressing stigma. Patient-focused materials, which included a patient handout and video [53] *, were designed to shape staff and patient attitudes by reframing drinking as a health issue and to decrease stigma and educate staff and patients on the spectrum of unhealthy alcohol use, recommended limits, and evidence-based approaches to treating AUD. Focus on shared decision-making. Management of AUD was reframed as shared decision-making, which is a patient-centered approach familiar to PC teams. PC providers and integrated behavioral health clinicians were trained to engage patients in shared decision-making, which included assessing patients’ medical conditions; eliciting patient concerns, goals, values, and perceptions; offering information on options (i.e., counseling, medications, and specialty alcohol treatment); and working with patients to support them in choosing their goals and treatment(s). Integrated behavioral health clinicians. Social workers were trained and supported in the engagement and management of patients with AUD and other substance use disorders, including assessment, MI skills, shared decision-making, and referral to treatment as appropriate [54] *.
**Strategy #2: Supporting PC Teams through Electronic Health Record (EHR) Decision Support**
Components:
EHR prompts for screening. Alerted medical assistants when a patient had not had behavioral health screening within the past year (7-item screening tool, including AUDIT-C). EHR prompts for brief intervention and AUD assessment. Based on AUDIT-C score, the EHR triggered visual prompts for medical assistants to give providers an alcohol handout and/or to ask the patient to complete a DSM-5 AUD Symptom Checklist (paper 11-item questionnaire). EHR prompts for missed AUD assessment. If AUD assessment was missed at the prior visit, prompts alerted medical assistants to give patients a DSM-5 AUD Symptom Checklist at their next in-person visit. Decision support for AUD diagnosis. EHR auto-totaled DSM-5 AUD symptoms and gave interpretive scores (none, mild, moderate, severe) to guide providers’ next steps and help facilitate AUD diagnosis. EHR prompts to initiate AUD treatment (piloted at Site 3 with three providers). If providers diagnose patients with new AUD (defined in Section 2.5.3 using codes used for U.S. national health plan quality measures) [52], providers were alerted to schedule patients to come back for a follow up visit to initiate care. EHR prompts AUD monitoring (piloted at two sites for several months). EHR alerts medical assistants to give patients with new AUD a monitoring tool that included the AUDIT-C and prompted providers to address AUD at visit.
**Strategy #3: Systematic Monitoring and Feedback of Performance Measures Including Alcohol Screening and AUD Assessment Rates**
Components:
Performance monitoring and quality improvement. Local implementation teams met with practice coaches and reviewed data at Plan-Do-Check-Adjust meetings based on a cadence determined by site leadership: Site 1 met monthly, Site 2 met monthly for 5 months and then every other week, and Site 3 had weekly meetings, dropping to every other week and then monthly meetings as processes stabilized and targets were met. Quality improvement meetings with leaders. Monthly Plan-Do-Check-Adjust meetings included local implementation teams, practice coaches, local and regional leadership, and behavioral health leaders, and researchers.

* indicates adapted strategies based on early formative evaluation during pilot or additional grant funding (for video).

**Table 2:** Demographics of patients with PC visits pre- and post-implementation of Behavioral Health Integration including the Program of Sustained Patient-centered Alcohol-related Care (SPARC).

Measure	Pre (%)	Post (%)	*p* Value ^a^
	(*N* = 32,295)	(*N* = 39,599)	
Male *	38.0	40.1	< 0.0001
Race **			< 0.0001
Asian	5.9	5.1	
Black	2.4	2.3	
Other/Multiracial	6.9	6.6	
White	82.1	83.1	
Unknown	2.8	2.9	
Hispanic *			0.022
No	92.2	92.1	
Yes	5.1	4.9	
Unknown	2.7	3	
Age ***, mean (IQR)	54.5 (40, 68)	55.4 (42, 68)	< 0.0001

*p* value obtained from Fisher’s exact test (*), analysis of variance (**), or Wilcoxon rank sum test (***). ^a^ Patients with visits in both the Pre and Post periods were excluded from these statistical tests due to these tests’ assumption of independence.

**Table 3:** Changes in rates of alcohol screening, AUD symptom assessment, new diagnosis, and treatment of AUDs Pre- versus Post-Behavioral Health Integration (which includes the SPARC program).

Measure	Pre (%)	Post (%)	*p* Value *
	(*N* = 32,295)	(*N* = 39,599)	
Screened for unhealthy alcohol use	8.9	62	<0.0001
Positive screen	2.2	17	<0.0001
High risk unhealthy alcohol use	0.31	1.4	<0.0001
Assessed for AUDs	0.012	0.75	<0.0001
AUD (2 + symptoms)	0.0062	0.40	<0.0001
New AUD diagnosis **	0.39	0.58	0.0002
AUD treatment among patients with new AUDs **			
Within 14 days	0.065	0.10	0.083
Within 30 days	0.087	0.14	0.034
Within 90 days	0.110	0.18	0.024

* *p* values calculated using Wald test from fitting Generalized Estimating Equations (GEE) and using robust variance estimator. ** New AUD diagnosis and AUD treatment defined in text.

## Supplementary Materials

The following are available online at www.mdpi.com/1660-4601/14/9/1030/s1.

## References

[1] Rehm J., Mathers C., Popova S., Thavorncharoensap M., Teerawattananon Y., Patra J. (2009). Global burden of disease and injury and economic cost attributable to alcohol use and alcohol-use disorders. Lancet.

[2] Saitz R. (2005). Clinical practice. Unhealthy alcohol use. N. Engl. J. Med..

[3] Bryson C.L., Au D.H., Sun H., Williams E.C., Kivlahan D.R., Bradley K.A. (2008). Alcohol screening scores and medication nonadherence. Ann. Intern. Med..

[4] Ahmed A.T., Karter A.J., Warton E.M., Doan J.U., Weisner C.M. (2008). The relationship between alcohol consumption and glycemic control among patients with diabetes: The Kaiser Permanente Northern California Diabetes Registry. J. Gen. Intern. Med..

[5] Jonas D.E., Garbutt J.C., Amick H.R., Brown J.M., Brownley K.A., Council C.L., Viera A.J., Wilkins T.M., Schwartz C.J., Richmond E.M. (2012). Behavioral counseling after screening for alcohol misuse in primary care: A systematic review and meta-analysis for the U.S. Preventive Services Task force. Ann. Intern. Med..

[6] Moyer V.A. (2013). Preventive services task force, screening and behavioral counseling interventions in primary care to reduce alcohol misuse: U.S. preventive services task force recommendation statement. Ann. Intern. Med..

[7] Latimer N., Guillaume L., Goyder E., Chilcott J., Payne N. Alcohol Use Disorders—Preventing Harmful Drinking. Screening and Brief Interventions: Cost Effectiveness Review. https://www.google.com.hk/url?sa=t&rct=j&q=&esrc=s&source=web&cd=3&ved=0ahUKEwjC8v-BrYPWAhXMKcAKHTNYBrEQFgg7MAI&url=https%3A%2F%2Farms.evidence.nhs.uk%2Fresources%2Fhub%2F1033186%2Fattachment&usg=AFQjCNGSYH_4_4YDuGWELWDZfTjcbc_8Ow.

[8] Fleming M.F., Barry K.L., Manwell L.B., Johnson K., London R. (1997). Brief physician advice for problem alcohol drinkers. A randomized controlled trial in community-based primary care practices. JAMA.

[9] Fleming M.F., Mundt M.P., French M.T., Manwell L.B., Stauffacher E.A., Barry K.L. (2000). Benefit-cost analysis of brief physician advice with problem drinkers in primary care settings. Med. Care.

[10] Fleming M.F., Mundt M.P., French M.T., Manwell L.B., Stauffacher E.A., Barry K.L. (2002). Brief physician advice for problem drinkers: Long-term efficacy and benefit-cost analysis. Alcohol Clin. Exp. Res..

[11] Maciosek M.V., Coffield A.B., Edwards N.M., Flottemesch T.J., Goodman M.J., Solberg L.I. (2006). Priorities among effective clinical preventive services results of a systematic review and analysis. Am. J. Prev. Med..

[12] Solberg L.I., Maciosek M.V., Edwards N.M. (2008). Primary care intervention to reduce alcohol misuse ranking its health impact and cost effectiveness. Am. J. Prev. Med..

[13] Jonas D.E., Amick H.R., Feltner C., Bobashev G., Thomas K., Wines R., Kim M.M., Shanahan E., Gass C.E., Rowe C.J. (2014). Pharmacotherapy for adults with alcohol use disorders in outpatient settings: A systematic review and meta-analysis. JAMA.

[14] Bouza C., Angeles M., Munoz A., Amate J.M. (2004). Efficacy and safety of naltrexone and acamprosate in the treatment of alcohol dependence: A systematic review. Addiction.

[15] Berglund M. (2005). A better widget? Three lessons for improving addiction treatment from a meta-analytical study. Addiction.

[16] Rosner S., Hackl-Herrwerth A., Leucht S., Lehert P., Vecchi S., Soyka M. (2010). Acamprosate for alcohol dependence. Cochrane Database Syst. Rev..

[17] Rosner S., Hackl-Herrwerth A., Leucht S., Vecchi S., Srisurapanont M., Soyka M. (2010). Opioid antagonists for alcohol dependence. Cochrane Database Syst. Rev..

[18] Dunn C., Deroo L., Rivara F.P. (2001). The use of brief interventions adapted from motivational interviewing across behavioral domains: A systematic review. Addiction.

[19] Mann K.V. (2004). The role of educational theory in continuing medical education: Has it helped us?. J. Contin. Educ. Health Prof..

[20] Glasner-Edwards S., Rawson R. (2010). Evidence-based practices in addiction treatment: Review and recommendations for public policy. Health Policy.

[21] (2011). National Institute for Health and Clinical Effectiveness. Alcohol-Use Disorders: Diagnosis, Assessment and Managment of Harmful Drinking and Alcohol Dependence.

[22] VA Office of Quality and Performance VA/DoD Clinical Practice Guideline for the Management of Substance Use Disorders. Version 2.0. http://www.healthquality.va.gov/guidelines/mh/sud/index.asp.

[23] National Quality Forum (2007). National Voluntary Consensus Standards for the Treatment of Substance Use Conditions: Evidence-Based Treatment Practices.

[24] Glass J.E., Bohnert K.M., Brown R.L. (2016). Alcohol screening and intervention among United States adults who attend ambulatory healthcare. J. Gen. Intern. Med..

[25] Oslin D.W., Lynch K.G., Maisto S.A., Lantinga L.J., McKay J.R., Possemato K., Ingram E., Wierzbicki M. (2014). A randomized clinical trial of alcohol care management delivered in Department of Veterans Affairs primary care clinics versus specialty addiction treatment. J. Gen. Intern. Med..

[26] McGlynn E.A., Asch S.M., Adams J.L., Keesey J., Hicks J., DeCristofaro A., Kerr E.A. (2003). The quality of health care delivered to adults in the United States. N. Engl. J. Med..

[27] Willenbring M.L., Massey S.H., Gardner M.B. (2009). Helping patients who drink too much: An evidence-based guide for primary care clinicians. Am. Fam. Physician.

[28] Watkins K., Pincus H.A., Tanielian T.L., Lloyd J. (2003). Using the chronic care model to improve treatment of alcohol use disorders in primary care settings. J. Stud. Alcohol.

[29] Saitz R., Larson M.J., Labelle C., Richardson J., Samet J.H. (2008). The case for chronic disease management for addiction. J. Addict. Med..

[30] Bradley K.A., Kivlahan D.R. (2014). Bringing patient-centered care to patients with alcohol use disorders. JAMA.

[31] Bradley K.A., Williams E.C., Achtmeyer C.E., Volpp B., Collins B.J., Kivlahan D.R. (2006). Implementation of evidence-based alcohol screening in the Veterans Health Administration. Am. J. Manag. Care.

[32] Lapham G.T., Achtmeyer C.E., Williams E.C., Hawkins E.J., Kivlahan D.R., Bradley K.A. (2012). Increased documented brief alcohol interventions with a performance measure and electronic decision support. Med. Care.

[33] Mertens J.R., Chi F.W., Weisner C.M., Satre D.D., Ross T.B., Allen S., Pating D., Campbell C.I., Lu Y.W., Sterling S.A. (2015). Physician versus non-physician delivery of alcohol screening, brief intervention and referral to treatment in adult primary care: The ADVISe cluster randomized controlled implementation trial. Addict. Sci. Clin. Pract..

[34] Chi F.W., Weisner C.M., Mertens J.R., Ross T.B., Sterling S.A. (2017). Alcohol brief intervention in primary care: Blood pressure outcomes in hypertensive patients. J. Subst. Abuse Treat..

[35] Williams E.C., Johnson M.L., Lapham G.T., Caldeiro R.M., Chew L., Fletcher G.S., McCormick K.A., Weppner W.G., Bradley K.A. (2011). Strategies to implement alcohol screening and brief intervention in primary care settings: A structured literature review. Psychol. Addict. Behav..

[36] Glass J.E., Kristjansson S.D., Bucholz K.K. (2013). Perceived alcohol stigma: Factor structure and construct validation. Alcohol. Clin. Exp. Res..

[37] Williams E.C., Achtmeyer C.E., Young J.P., Rittmueller S.E., Ludman E.J., Lapham G.T., Lee A.K., Chavez L.J., Berger D., Bradley K.A. (2016). Local implementation of alcohol screening and brief intervention at five veterans health administration primary care clinics: Perspectives of clinical and administrative staff. J. Subst. Abuse Treat..

[38] McCormick K.A., Cochran N.E., Back A.L., Merrill J.O., Williams E.C., Bradley K.A. (2006). How primary care providers talk to patients about alcohol: A qualitative study. J Gen Intern Med..

[39] Spandorfer J.M., Israel Y., Turner B.J. (1999). Primary care physicians’ views on screening and management of alcohol abuse: Inconsistencies with national guidelines. J. Fam. Pract..

[40] Bradley K.A., Williams E.C., Ries R.K., Miller S.C., Fiellin D.A., Saitz R. (2013). Implementation of SBI in Clinical Settings Using Quality Improvement Principles. Principles of Addiction Medicine.

[41] Bradley K.A., Lapham G.T., Hawkins E.J., Achtmeyer C.E., Williams E.C., Thomas R.M., Kivlahan D.R. (2011). Quality concerns with routine alcohol screening in VA clinical settings. J. Gen. Intern. Med..

[42] Chavez L.J., Williams E.C., Lapham G.T., Rubinsky A.D., Kivlahan D.R., Bradley K.A. (2016). Changes in patient-reported alcohol-related advice following veterans health administration implementation of brief alcohol interventions. J. Stud. Alcohol. Drugs.

[43] Berger D., Lapham G.T., Shortreed S., Hawkins E.J., Rubinsky A.D., Williams E.C., Achtmeyer C.E., Kivlahan D.R., Bradley K.A. (2017). Increasing rates of documented alcohol advice: More advice or just more documentation?. J. Gen. Intern. Med..

[44] Reynolds C.F., Frank E. (2016). US Preventive services task force recommendation statement on screening for depression in adults: Not good enough. JAMA Psychiatry.

[45] Kroenke K., Spitzer R.L. (2002). The PHQ-9: A new depression diagnostic and severity measure. Psychiatr. Ann..

[46] Lowe B., Schenkel I., Carney-Doebbeling C., Gobel C. (2006). Responsiveness of the PHQ-9 to psychopharmacological depression treatment. Psychosomatics.

[47] Bradley K.A., DeBenedetti A.F., Volk R.J., Williams E.C., Frank D., Kivlahan D.R. (2007). AUDIT-C as a brief screen for alcohol misuse in primary care. Alcohol Clin. Exp. Res..

[48] Lapham G., Lee A., Caldeiro R., McCarty D., Browne K., Walker D., Kivlahan D., Bradley K. (2017). Frequency of cannabis use among primary care patients in Washington state where use is legal. J. Am. Board Fam. Med..

[49] Smith P.C., Schmidt S.M., Allensworth-Davies D., Saitz R. (2010). A single-question screening test for drug use in primary care. Arch. Intern. Med..

[50] National Institute on Alcohol Abuse and Alcoholism (2005). Helping Patients Who Drink Too Much: A Clinician’s Guide (Updated 2005 Edition).

[51] Whitlock E.P., Polen M.R., Green C.A., Orleans T., Klein J. (2004). Behavioral counseling interventions in primary care to reduce risky/harmful alcohol use by adults: A summary of the evidence for the U.S. Preventive Services Task Force. Ann. Intern. Med..

[52] National Committee for Quality Assurance (2011). HEDIS 2011 Technical Specifications.

[53] Bradley K.A. Preparing Clinicians and Patients for Shared Decision-making about Alcohol Use Disorders: Development of an Entertaining Video (The Mike Evans Video Project).

[54] Bradley K.A., Caldeiro R. Alcohol and Drug Use Disorders: 3 Visits in 30 Days (The 3:30 Project).

[55] Whooley M.A., Avins A.L., Miranda J., Browner W.S. (1997). Case finding instruments for depression. Two questions are as good as many. J. Gen. Intern. Med..

[56] Rubinsky A.D., Dawson D.A., Williams E.C., Kivlahan D.R., Bradley K.A. (2013). AUDIT-C scores as a scaled marker of mean daily drinking, alcohol use disorder severity, and probability of alcohol dependence in a U.S. general population sample of drinkers. Alcohol Clin. Exp. Res..

[57] Rubinsky A.D., Kivlahan D.R., Volk R.J., Maynard C., Bradley K.A. (2010). Estimating risk of alcohol dependence using alcohol screening scores. Drug Alcohol Depend..

[58] Johnson J.A., Lee A., Vinson D., Seale J.P. (2013). Use of AUDIT-based measures to identify unhealthy alcohol use and alcohol dependence in primary care: A validation study. Alcohol Clin. Exp. Res..

[59] Hasin D.S., O’Brien C.P., Auriacombe M., Borges G., Bucholz K., Budney A., Compton W.M., Crowley T., Ling W., Petry N.M. (2013). DSM-5 criteria for substance use disorders: Recommendations and rationale. Am. J. Psychiatry.

[60] Bradley K.A., Chavez L.J., Lapham G.T., Williams E.C., Achtmeyer C.E., Rubinsky A.D., Hawkins E.J., Saitz R., Kivlahan D.R. (2013). When quality indicators undermine quality: Bias in a quality indicator of follow-up for alcohol misuse. Psychiatr. Serv..

[61] Zeger S.L., Liang K.Y. (1986). Longitudinal data analysis for discrete and continuous outcomes. Biometrics.

[62] Stetler C.B., Legro M.W., Wallace C.M., Bowman C., Guihan M., Hagedorn H., Kimmel B., Sharp N.D., Smith J.L. (2006). The role of formative evaluation in implementation research and the QUERI experience. J. Gen. Intern. Med..

[63] King N., Symon G., Cassell C. (1998). Template Analysis. Qualitative Methods and Analysis in Organizational Research.

[64] Greenhalgh T., Robert G., Macfarlane F., Bate P., Kyriakidou O. (2004). Diffusion of innovations in service organizations: Systematic review and recommendations. Milbank Q..

[65] Batalden P.B., Nelson E.C., Edwards W.H., Godfrey M.M., Mohr J.J. (2003). Microsystems in health care: Part 9. Developing small clinical units to attain peak performance. Jt. Comm. J. Qual. Saf..

[66] Elwyn G., Dehlendorf C., Epstein R.M., Marrin K., White J., Frosch D.L. (2014). Shared decision making and motivational interviewing: Achieving patient-centered care across the spectrum of health care problems. Ann. Fam. Med..

[67] Wallace P., Cutler S., Haines A. (1988). Randomised controlled trial of general practitioner intervention in patients with excessive alcohol consumption. BMJ.

[68] Coulehan J.L., Zettler-Segal M., Block M., McClelland M., Schulberg H.C. (1987). Recognition of alcoholism and substance abuse in primary care patients. Arch. Intern. Med..

[69] Buchsbaum D.G., Buchanan R.G., Poses R.M., Schnoll S.H., Lawton M.J. (1992). Physician detection of drinking problems in patients attending a general medicine practice. J. Gen. Intern. Med..

[70] Barnes H.N., Aronson M.D., Delbanco T.L. (1987). Alcoholism—A Guide for the Primary Care Physician.

[71] Buchsbaum D.G., Buchanan R.G., Lawton M.J., Elswick R.K., Schnoll S.H. (1993). A program of screening and prompting improves short-term physician counseling of dependent and nondependent harmful drinkers. Arch. Intern. Med..

[72] Grant B.F., Goldstein R.B., Saha T.D., Chou S.P., Jung J., Zhang H., Pickering R.P., Ruan W.J., Smith S.M., Huang B. (2015). Epidemiology of DSM-5 alcohol use disorder: Results from the national epidemiologic survey on alcohol and related conditions III. JAMA Psychiatry.

