# Supplementary material for: Evaluation of a Pilot Implementation to Integrate Alcohol-Related Care within Primary Care
Source: Int J Environ Res Public Health. 2017 Sep 8;14(9):1030. doi: 10.3390/ijerph14091030 (PMC5615567; doi:10.3390/ijerph14091030)
Supplement: Supplementary file 1 [file ijerph-14-01030-s001.zip › KP_Alcohol_Video_bro_internal_170208.pdf]

How our understanding has changed

Old Stereotypes<br>**In the past experts thought…**	New Knowledge<br>**Now experts know…**
People who were not “alcoholic” did not need to watch how much they drank.	Drinking can cause problems for anyone. So we focus on preventing these problems by educating everyone about alcohol use.
Alcoholism was due to a lack of will power. It was not generally treated by doctors.	An alcohol use disorder is a brain condition caused by many factors, including how much a person drinks.
Doctors had to wait until people with alcoholism wanted help.	Asking people about their alcohol use and giving them advice about it is part of high-quality health care for everyone.
There was a “one-size-fits-all” approach to alcohol treatment–and we only offered people group treatment based on the 12 steps of Alcoholics Anonymous (AA).	People with alcohol use disorders can choose from several proven treatment options:<br>• Individual or couples counseling<br>• Group counseling<br>• Medications<br>• Mutual help programs like SMART Recovery or AA

What are the recommended limits?*

MEN

Per day: No more than 2 drinks on average, and no more than 4 drinks on any day

Per week: No more than 14 drinks total

WOMEN

Per day: No more than 1 drink on average, and no more than 3 drinks on any day

Per week: No more than 7 drinks total

1 Drink =

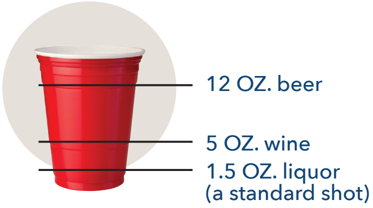

Drinking above these limits increases your risk of:

- Weight gain
- Insomnia
- Forgetting medications
- Medication interactions
- Surgical complications
- High blood pressure
- Depression and anxiety
- Liver or pancreatic disease
- Bleeding from the stomach
- Stroke
- Dementia
- Seizures
- Breast, prostate, colon and other cancers
- Heart disease, including heart failure
- Death

*Experts recommend no alcohol use for women who are pregnant, people who have liver disease, or people who have had problems due to drinking in the past.

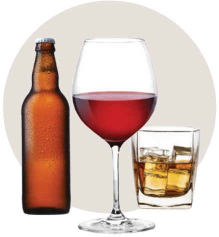

Moving beyond stereotypes

Experts no longer view drinking alcohol as a black and white issue, where people are either “alcoholic” or not. Instead, we use the term “alcohol use disorders” to describe a broad range of problems related to drinking.

Experts have also stopped recommending that people drink for their health. Why? Because the health and social problems that drinking can cause far outweigh any potential health benefits.

Did you know

- About 1 in 4 adults drinks more alcohol than is recommended for good health. And about 1 in 12 has an alcohol use disorder.
- People who drink above recommended limits are at risk for a variety of health problems.
- The risk of death increases in women who have more than 7 drinks per week and in men who have more than 14 drinks per week.

Talk With Your Doctor

Even if you don’t want to stop drinking, treatment can still help you cut back. Ask yourself these important questions, then talk with your doctor about your answers.

- ☐ Have you had times when you drank more, or for longer, than you wanted to?
- ☐ Have you wanted to cut back or stop drinking more than once, but found that you couldn’t?
- ☐ Do you spend a lot of time drinking or feeling hung-over?
- ☐ Do you feel an urge to drink or a craving for alcohol?
- ☐ Has drinking or feeling hung-over made it harder for you to take care of your responsibilities?
- ☐ Have you continued to drink even when it was causing trouble with your family or friends?
- ☐ Have you stopped doing things you enjoy because of your drinking?
- ☐ Do you ever do dangerous things after drinking, such as drive a car or have unsafe sex?
- ☐ Have you continued to drink even when it made you feel depressed or anxious or caused other health problems?
- ☐ Do you need to drink more than you used to to feel the effect you want?
- ☐ Do you feel like you’re not yourself when you don’t drink—for example, do you feel irritable, have trouble sleeping, or notice other problems?

A ReThink of the Way we Drink
https://youtu.be/tbKbq2lytC4

KAISER PERMANENTE®

DL-108167
2015259

Alcohol and health...

What you should know

KAISER PERMANENTE®
