# Supplementary figures and images for: Evaluation of a Pilot Implementation to Integrate Alcohol-Related Care within Primary Care
Source: Int J Environ Res Public Health. 2017 Sep 8;14(9):1030. doi: 10.3390/ijerph14091030 (PMC5615567; doi:10.3390/ijerph14091030)

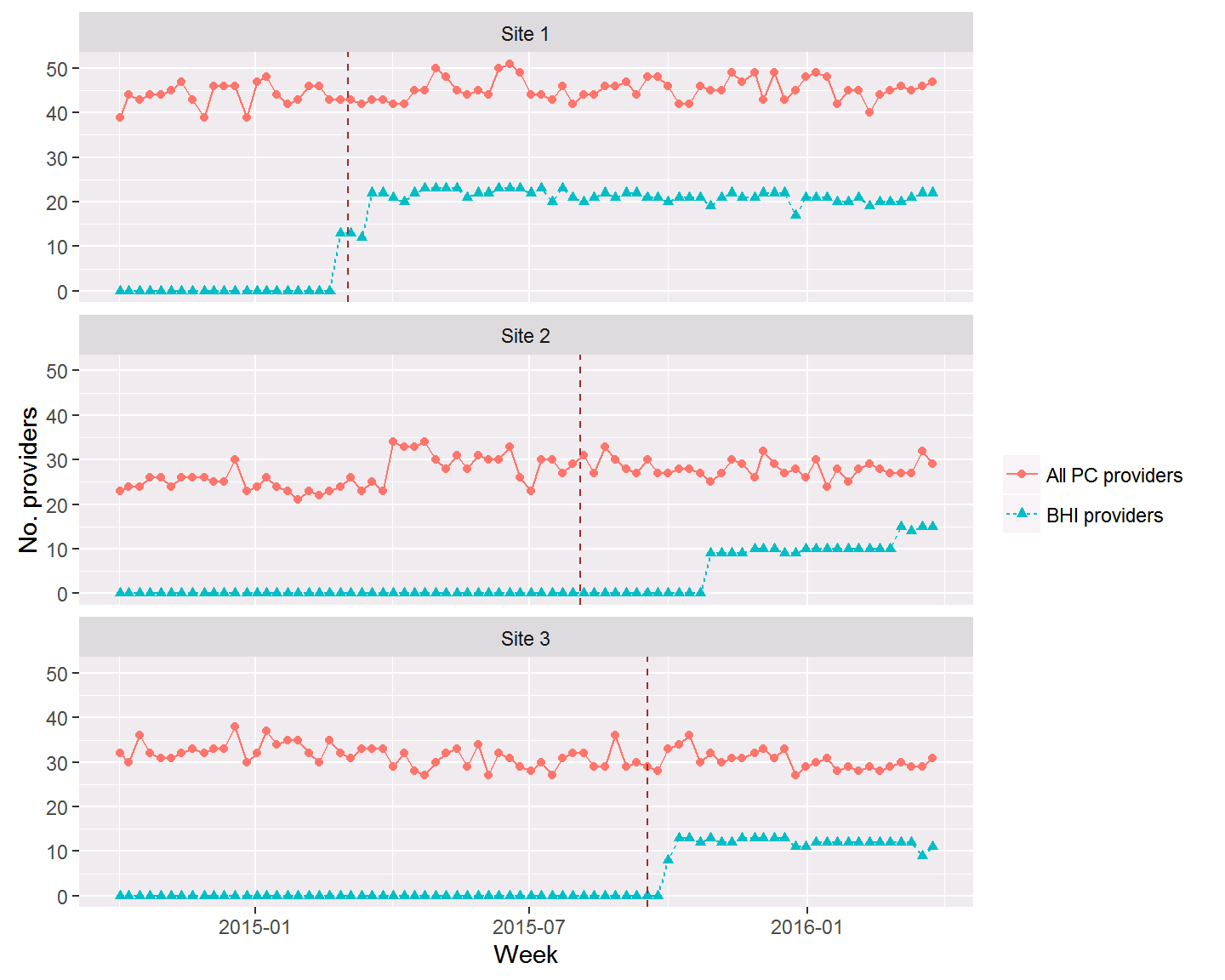

Supplement: Supplementary file 1 [file ijerph-14-01030-s001.zip › FigureS1.png]
